# Vesnarinone and glucocorticoids cooperatively induce G_1_ arrest and have an anti-tumour effect on human non-small cell lung carcinoma cells grown in nude mice

**DOI:** 10.1038/sj.bjc.6690327

**Published:** 1999-04

**Authors:** Y Honma, Y Yamamoto-Yamaguchi, Y Kanatani

**Affiliations:** Department of Chemotherapy, Saitama Cancer Center Research Institute, 818 Komuro, Ina, Saitama 362, Japan

**Keywords:** vesnarinone, glucocorticoid, lung carcinoma, xenograft, differentiation

## Abstract

Vesnarinone, an oral cardiotonic, inhibited the growth of several human non-small cell lung carcinoma cell lines, and its anti-proliferative effects in vitro and in vivo were greatly enhanced by combination with glucocorticoids, but not other steroids. Simultaneous treatment with vesnarinone and dexamethasone is the most effective to evoke the synergistic effect in the growth inhibition of lung carcinoma EBC-1 cells. Dexamethasone and other glucocorticoids induced morphological changes in EBC-1 cells and these agents together with vesnarinone induced alkaline phosphatase activity, which is a typical marker of type II pneumocyte maturation. This treatment arrested the growth of the cells at the G_1_ phase, indicating that this treatment is cytostatic rather than cytotoxic. These results suggest that vesnarinone plus glucocorticoid might be useful in lung cancer therapy. © 1999 Cancer Research Campaign


*British Journal of Cancer* (1999) **80**(1/2), 96–103
© 1999 Cancer Research Campaign
Article no. bjoc.1998.0327

### Vesnarinone and glucocorticoids cooperatively induce $G_1$ arrest and have an anti-tumour effect on human non-small cell lung carcinoma cells grown in nude mice

**Y Honma, Y Yamamoto-Yamaguchi and Y Kanatani**

Department of Chemotherapy, Saitama Cancer Center Research Institute, 818 Komuro, Ina, Saitama 362, Japan

**Summary** Vesnarinone, an oral cardiotonic, inhibited the growth of several human non-small cell lung carcinoma cell lines, and its anti-proliferative effects in vitro and in vivo were greatly enhanced by combination with glucocorticoids, but not other steroids. Simultaneous treatment with vesnarinone and dexamethasone is the most effective to evoke the synergistic effect in the growth inhibition of lung carcinoma EBC-1 cells. Dexamethasone and other glucocorticoids induced morphological changes in EBC-1 cells and these agents together with vesnarinone induced alkaline phosphatase activity, which is a typical marker of type II pneumocyte maturation. This treatment arrested the growth of the cells at the $G_1$ phase, indicating that this treatment is cytostatic rather than cytotoxic. These results suggest that vesnarinone plus glucocorticoid might be useful in lung cancer therapy.

**Keywords:** vesnarinone; glucocorticoid; lung carcinoma; xenograft; differentiation

Currently, lung cancer is a leading cause of cancer-related adult deaths, and its incidence continues to rise. The overall response rate of non-small cell lung cancer (NSCLC) is in the range of 20–30% with 3–5% of complete responses (Donnadieu et al, 1991). The current results of treatment for NSCLC clearly call for improvements.

Vesnarinone (3,4-dihydro-6-[4-(3,4-dimethoxybenzoyl-1-piperazinyl]-2(1H)-quinolinone) is an oral inotropic agent which has been used for the treatment of chronic heart failure in Japan (OPC-8212 Multicenter Research Group, 1990). In addition to this inotropic activity, we and others have shown that vesnarinone has differentiation-inducing activity in several human haematopoietic and non-haematopoietic malignant cells (Sato et al, 1995, 1996). Although the mechanism of differentiation induced by vesnarinone remains unknown, vesnarinone, alone or in combination with other differentiation inducers, can significantly induce or enhance the differentiation of myelomonocytic leukaemia cells (Sato et al, 1996). Vesnarinone is effective when administered perorally, and the serum level of vesnarinone can be obtained up to 30 $\mu$g ml$^{-1}$ (Miyamoto and Sasabe, 1984; Taira et al, 1984). Treatment with 30 $\mu$g ml$^{-1}$ of vesnarinone markedly enhanced the morphological differentiation of HL-60 and U937 cells induced by suboptimal concentrations of some differentiation inducers, including retinoic acid (Sato et al, 1996). The pharmacokinetics and toxicity of vesnarinone have already been studied (Miyamoto and Sasabe, 1984; Taira et al, 1984) and it has been approved for use as a cardiotonic drug. These results suggest that combined treatment with vesnarinone and other non-toxic drugs may be a new potent therapy for some types of malignancies.

*Received 17 December 1998*
*Revised 26 August 1998*
*Accepted 27 August 1998*

*Correspondence to:* Y Honma

Cell cycle checkpoints represent a new set of potential targets for chemotherapeutic compounds (Hartwell and Kastan, 1994; Karp and Broder, 1995). Physiological and/or non-genotoxic compounds that induce cell cycle arrest and/or apoptosis in cancer cells should be useful for cancer chemotherapy, since DNA-damaging agents produce serious side-effects in the host and sometimes induce secondary leukaemia and tumours. Therefore, it is worthwhile to search for less toxic drugs that have been used in clinical trials for other diseases, to develop a new therapy for malignancies.

In the present study, we examined the growth-inhibitory effect of vesnarinone on human non-small lung carcinoma cell lines in combination with various compounds, and found that vesnarinone and glucocorticoids had synergistic on the growth of lung carcinoma cells in vitro and in vivo.

### MATERIALS AND METHODS

### Chemicals

Vesnarinone was obtained from Otsuka Pharmaceutical Company (Tokushima, Japan), dissolved in dimethyl sulphoxide (DMSO) and diluted with phosphate-buffered saline (PBS). Dexamethasone (Dex), 3-(4,5-dimethylthiazol-2-yl)-2,5-diphenyltetrazolium bromide (MTT), *p*-nitrophenyl phosphate, RNase A and propidium iodide were obtained from Sigma Chemical Co. (St Louis, MO, USA). DMSO and all other chemicals were obtained from Wako Pure Chemicals (Osaka, Japan).

### Cells and cell culture

Human lung carcinoma EBC-1, LK2, PC9, ABC-1 and A549 cell lines were maintained in RPMI-1640 medium (GIBCO, Great Island, NY, USA) supplemented with 10% heat-inactivated fetal bovine serum and non-essential amino acids of modified Eagle's medium (MEM) at 37°C in a humidified atmosphere of 5% $CO_2$ in air (Goto et al, 1994, 1996). The cell lines were kindly supplied from the Japanese Cancer Research Resources Bank (Tokyo, Japan).

### Assay of cell growth

The cells were seeded at a concentration of $2 \times 10^4$ ml$^{-1}$ in a multi-dish (Nunc, Roskilde, Denmark). After culture with or without the test compounds for 7 days, viable cells were examined by MTT assay (Goto et al, 1994). Briefly, 100 µl of MTT solution (5 mg ml$^{-1}$ in PBS) were added to each well. After incubation with MTT for 4 h, the cells were centrifuged at 1000 ***g*** for 10 min. The precipitates were dissolved in 1 ml of DMSO and their absorptions at 560 nm were determined.

### Assay of the cumulative cell number

The cell density of untreated cells was kept at $1–10 \times 10^5$ ml$^{-1}$ to maintain continuous logarithmic growth. The cells were collected by treatment with 0.1% trypsin–0.04% EDTA (GIBCO, Grand Island, NY, USA) for 5 min with gentle shaking, and were transferred every 3 days. The cell density of the treated cells was kept at $3–8 \times 10^5$ ml$^{-1}$ to maintain cells in growing phase, and transferred every 3 or 6 days by the trypsinization. The treatment with trypsin did not affect the cell morphology and adhesiveness. The cell number was counted with a Coulter Counter (Model ZBI; Coulter Electronics, Hialeah, FL, USA). The cumulative cell number was calculated from the counts and dilution used when feeding the culture.

### Assay of alkaline phosphatase activity

Alkaline phosphatase activity is a marker for the maturation of type II pneumocytes and is not expressed in other alveolar cells (Edelson et al, 1988; McCormick et al, 1995). Cells were washed twice with cold PBS, resuspended to a cell density of $10^7$ cells ml$^{-1}$ of cold distilled water, and sonicated for 10 s. The reaction mixture contained 50 mM glycine–NaOH (pH 10.5), 0.5 mM magnesium chloride, 4.2 mM *p*-nitrophenyl phosphate, and the cell lysate in a total volume of 0.4 ml. The incubation was allowed to proceed for 60 min at 37°C, and the reaction was then stopped by adding of 1 ml of 0.1 M NaOH. The absorbance of *p*-nitrophenol was determined at 410 nm to calculate enzyme activity.

### Cell cycle analysis

The cell cycle was analysed using propidium iodide-stained nuclei. Samples of $2 \times 10^6$ cells were harvested at the time points indicated, washed in ice-cold PBS, fixed by the addition of 100% ethanol and left for 30 min on ice. The cell pellet was washed and suspended in 200 µl of 1.12% sodium citrate containing RNase A (250 µg ml$^{-1}$) for 30 min at room temperature. Thereafter, the cells were stained with 50 µg ml$^{-1}$ of propidium iodide in the presence of 1.12% sodium citrate and analysed in a fluorescence-activated cell sorter.

### Western blot analysis of Rb protein

Cells were washed once with cold PBS, lysed by sonication for 30 s at a concentration of $2 \times 10^7$ cells ml$^{-1}$ in sample buffer, and then resolved on 7.5% sodium dodecyl sulphate polyacrylamide gels (SDS-PAGE) as described by Laemmli (1970). The proteins were transferred electrophoretically from the gel to an Immobilon-P membrane (Millipore) and immunoblotted with anti-Rb protein (Santa Cruz Biotechnology, Inc., Santa Cruz, CA, USA). The proteins were quantified by an enzyme-linked immunosorbent assay as described previously (Kanatani et al, 1993).

### Transplantation of lung carcinoma cells into nude mice

Female athymic nude mice with a BALB/c genetic background were supplied by CLEA Japan (Tokyo, Japan). The in vivo experiments were performed in accordance with the guidelines of our institute (Guide for Animal Experimentation, Saitama Cancer Center). Mice were inoculated subcutaneously (s.c.) with $10^6$ EBC-1 or PC9 cells. Mice were given a daily intraperitoneal (i.p.) injection of 0.2 ml of PBS including prednisolone (300 mg kg$^{-1}$) and/or peroral administration of vesnarinone (5 mg kg$^{-1}$), with the first injection given 4 days after the inoculation of tumour cells. Tumour size was measured with vernier calipers every day. Statistical analysis was performed using Student's *t*-test.

### RESULTS

### Combined effects of vesnarinone and Dex on the growth of lung carcinoma cell lines

To measure the growth-inhibitory effects of drugs on lung carcinoma cells, the number of viable cells was determined by the MTT assay after 7 days of exposure to various concentrations of drugs. The growth-inhibitory effects of drugs were examined by determining the concentrations of drugs required to reduce the cell number to one-half that of untreated cells ($IC_{50}$). Vesnarinone inhibited the growth of lung carcinoma cells in a dose-dependent manner, and the $IC_{50}$ of vesnarinone in squamous cell carcinoma LK2 cells was lower than 10 µg ml$^{-1}$. However, the growth of the other NSCLC cells was less sensitive, and the respective $IC_{50}$s were more than 20 µg ml$^{-1}$ ($IC_{50}$: 23.4, 21.3, 29.7 and 30.6 µg ml$^{-1}$ in EBC-1, PC9, ABC-1 and A549 cells respectively). Vesnarinone

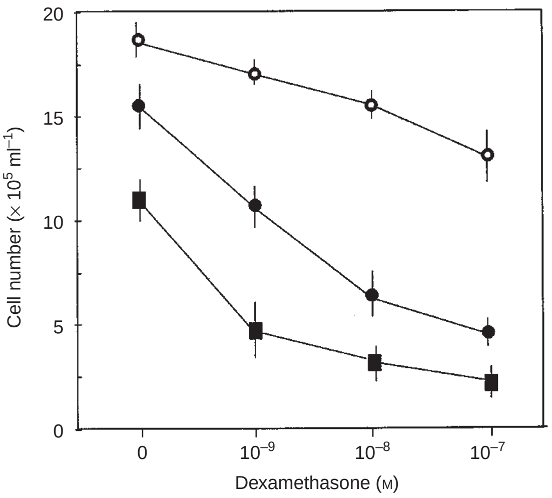

**Figure 1** Effects of Dex and vesnarinone on the growth of EBC-1 cells. Cells were cultured with various concentrations of Dex in the presence of 0 (○), 10 (●), or 20 (■) µg ml$^{-1}$ of vesnarinone for 7 days. Values are means ± s.d. of four determinations

© Cancer Research Campaign 1999

**Table 1** Effects of various steroids on the growth of EBC-1 cells in the presence of vesnarinone

| Steroid ($10^{-8}$ M) | Cell number (% of control) | |
|---|---|---|
| | –Vesnarinone | +Vesnarinone |
| None | 100 | 86.2 ± 3.7 |
| Dexamethasone | 92.8 ± 4.9 | 38.9 ± 2.1 |
| Prednisolone | 95.4 ± 5.2 | 39.5 ± 3.2 |
| Hydrocortisone | 98.8 ± 5.5 | 44.6 ± 3.4 |
| Corticosterone | 99.3 ± 5.2 | 56.8 ± 3.6 |
| 11-Epicortisol | 99.2 ± 5.6 | 82.3 ± 4.2 |
| Progesterone | 93.9 ± 4.9 | 83.9 ± 3.9 |
| 11β-Estradiol | 98.4 ± 5.1 | 84.7 ± 4.3 |
| Testosterone | 99.1 ± 4.7 | 86.0 ± 3.5 |

Cells were treated with various steroids in the presence or absence of 10 μg ml$^{-1}$ vesnarinone for 7 days, and the number of viable cells was determined by MTT assay. Data represent means from three experiments ± s.d.

**Table 2** Combined effects of Dex and vesnarinone on the growth of EBC-1 cells

| Regimen | Days 1–4 | Days 5–8 | Growth inhibition (%) |
|---|---|---|---|
| 1–1 | None | None | 0 ± 0.1 |
| 1–2 | | Dex | 4.2 ± 0.2 |
| 1–3 | | Ves | 33.3 ± 1.9 |
| 1–4 | | Dex+Ves | 44.2 ± 2.3 |
| 2–1 | Dex | None | 3.1 ± 0.2 |
| 2–2 | | Dex | 2.9 ± 0.2 |
| 2–3 | | Ves | 36.2 ± 2.5 |
| 3–1 | Ves | None | 13.2 ± 1.4 |
| 3–2 | | Dex | 27.8 ± 1.8 |
| 3–3 | | Ves | 46.8 ± 3.3 |
| 4–1 | Dex+Ves | None | 52.6 ± 3.5 |
| 4–2 | | Dex+Ves | 85.3 ± 4.1 |

Cells were cultured for 4 days in the presence or absence of 10 nM Dex, 10 μg ml$^{-1}$ vesnarinone (Ves), or 10 nM Dex plus 10 μg ml$^{-1}$ Ves. On day 4, the cultures were washed with fresh medium, and the cultures reincubated in the presence or absence of the same concentration of Dex or Ves. Means ± s.d. of three determinations.

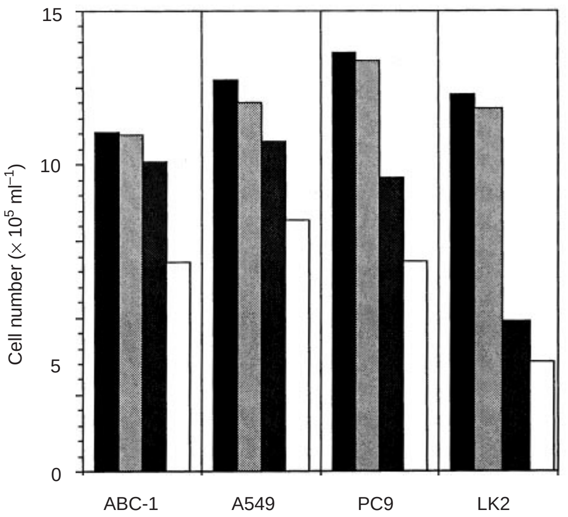

**Figure 2** Effects of Dex and vesnarinone on the growth of human lung cancer cells. Cells were cultured with $10^{-9}$ M Dex (□), 10 μg ml$^{-1}$ vesnarinone (■), and $10^{-9}$ M Dex plus 10 μg ml$^{-1}$ vesnarinone (□) for 7 days. ■, untreated cells. Values are means of three determinations

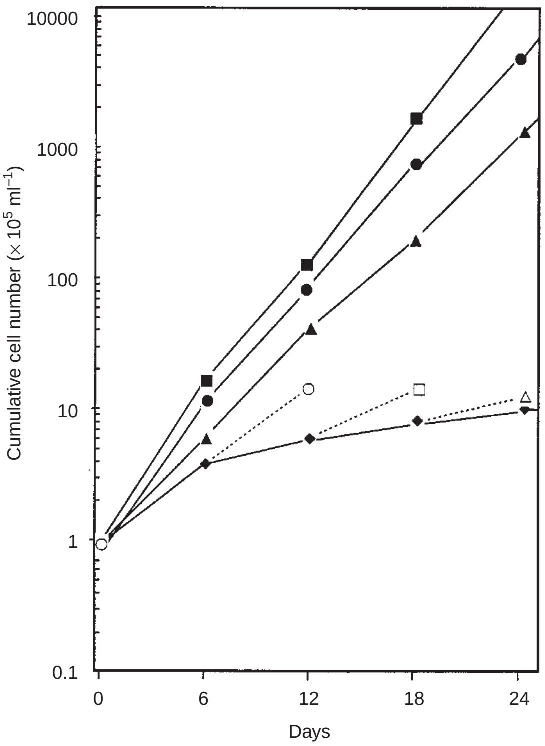

**Figure 3** Proliferation of EBC-1 cells in long-term culture with Dex and vesnarinone. Cells were cultured without (■) or with 2 × $10^{-8}$ M Dex (●), 10 μg ml$^{-1}$ vesnarinone (▲), or 2 × $10^{-8}$ M Dex plus 10 μg ml$^{-1}$ of vesnarinone (◆). Open symbols, cells were washed and then cultured without drugs. Cells were treated with Dex plus vesnarinone for 6 (○), 12 (□) and 18 (△) days, and then washed and reincubated without drugs for further 6 days. Data are means of three separate experiments is effective when administered perorally, and the serum level of vesnarinone can be obtained up to 30 μg ml$^{-1}$ (Miyamoto and Sasabe, 1984; Taira et al, 1984). These results suggest that treatment with vesnarinone alone is clinically effective in only a limited number of cases of NSCLC.

Next, we examined the growth-inhibitory effect of various compounds in combination with vesnarinone. Of the compounds tested, Dex more than additively enhanced the vesnarinone-induced inhibition of the growth of lung carcinoma EBC-1 cells (Figure 1). To determine the structure required for the growth inhibition, the effect of various steroids was examined. The growth inhibition in the presence of vesnarinone was closely correlated with glucocorticoid activity (Table 1). The growth-inhibitory effect of vesnarinone was not enhanced by retinoic acid ($10^{-9}$–$10^{-6}$ M), 1 α, 25-dihydroxyvitamin $D_3$ ($10^{-9}$–$10^{-5}$ M), sodium butyrate (0.1–2.5 mM), interferon-α (1–3000 IU ml$^{-1}$), actinomycin D

© Cancer Research Campaign 1999

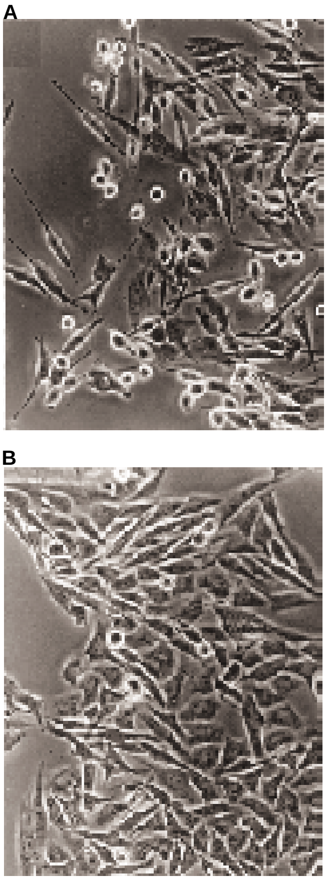

**Figure 4** Morphological changes in human lung carcinoma EBC-1 cells by Dex plus vesnarinone. Cells were treated without (**A**) or with (**B**) $2 \times 10^{-8}$ M Dex for 2 days. Original magnification $\times$ 400

(0.2–16 nM), cytosine arabinoside ($10^{-10}$–$10^{-8}$ M), or doxorubicin (1–50 nM) (data not shown). The synergistic effect of vesnarinone and Dex was observed in the growth of all of the NSCLC cells tested (Figure 2).

The effect of the time of the addition of Dex or vesnarinone on growth inhibition was examined. Pretreatment with Dex did not affect the growth-inhibitory activity of vesnarinone plus Dex, and the delayed addition of Dex also did not affect growth inhibition (data not shown). Table 2 indicates that simultaneous treatment is the most effective to evoke the synergistic effect of vesnarinone and Dex.

The effect of continuous treatment with 10 µg ml$^{-1}$ of vesnarinone and $2 \times 10^{-8}$ M Dex added simultaneously on the proliferation of EBC-1 cells was examined for 24 days. The cell density was kept at $1–10 \times 10^{5}$ ml$^{-1}$ to maintain cells in the growing phase, and the cumulative cell number was calculated from the counts and dilution used when feeding the culture. Continuous treatment with 10 µg ml$^{-1}$ of vesnarinone or $2 \times 10^{-8}$ M Dex only slightly inhibited the growth of EBC-1 cells. However, combined treatment with vesnarinone and Dex caused significant growth inhibition (Figure 3). When the cells were treated for 18 days, and then washed and cultured without the drugs, cell growth was greatly inhibited at day 24, indicating that the growth-inhibitory effect of vesnarinone and Dex was irreversible in long-term culture. These results suggest that the combined treatment has therapeutic value in the chemotherapy of some lung cancers.

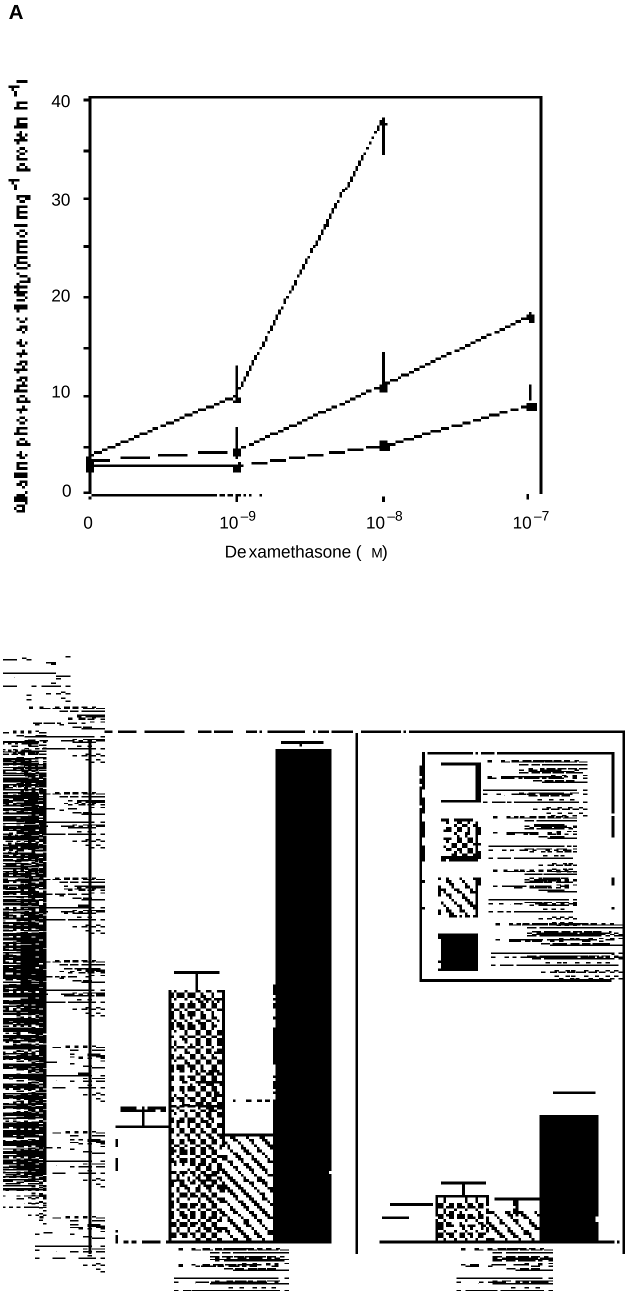

**Figure 5** Induction of alkaline phosphatase activity in lung carcinoma cells by Dex in combination with vesnarinone. (**A**) EBC-1 cells were treated with Dex in the presence of 0 (■), 10 (●), or 20 (▲) µg ml$^{-1}$ of vesnarinone for 7 days. (**B**) ABC-1 and PC9 cells were treated with $10^{-8}$ M Dex and/or 10 µg ml$^{-1}$ of vesnarinone for 7 days. Values are means ± s.d. of four determinations

### Effects of Dex and vesnarinone on morphological changes and alkaline phosphatase activity of EBC-1 cells

Dex induced morphological changes in EBC-1 cells, although the growth of EBC-1 cells was not essentially affected by even a high concentration of Dex. Untreated EBC-1 cells were spindle-shaped, while Dex-treated cells adhered closely to each other and were cuboidal and polygonal (Figure 4). Vesnarinone alone did not

*© Cancer Research Campaign 1999*

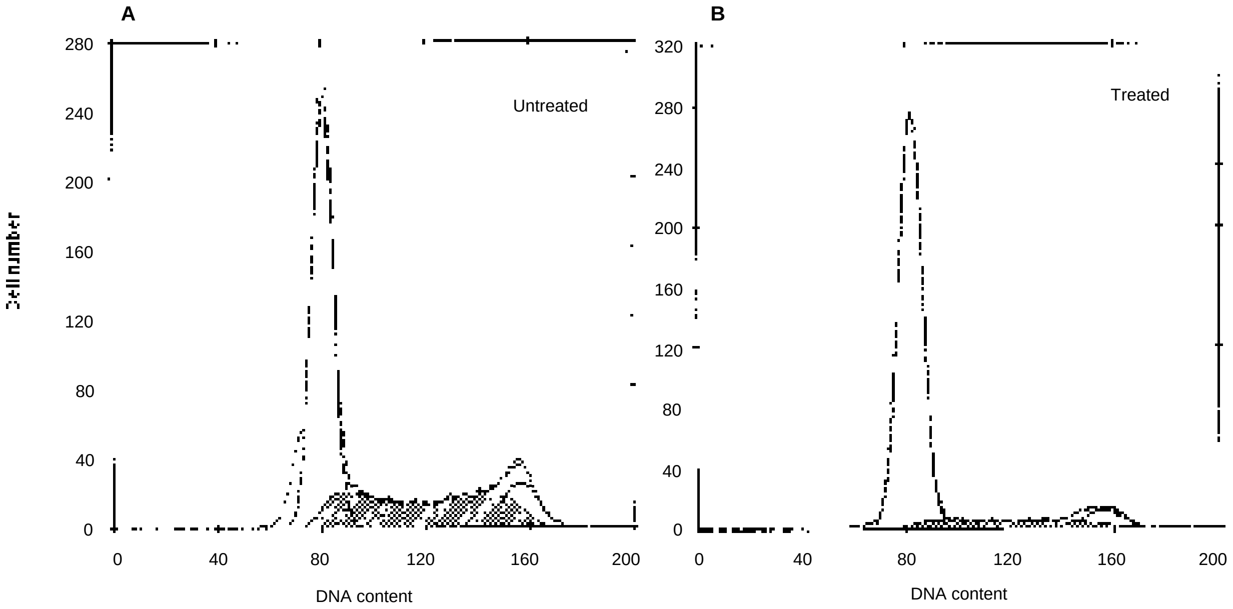

**Figure 6** Induction of $G_1$ arrest in EBC-1 cells treated with Dex plus vesnarinone. Cells were cultured without (**A**) or with (**B**) $2 \times 10^{-8}$ M Dex plus 10 µg $ml^{-1}$ of vesnarinone for 4 days, and DNA histograms were then analysed

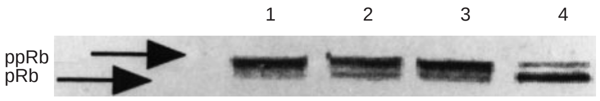

**Figure 7** Western blot for Rb protein from EBC-1 cells treated with Dex and vesnarinone. Cells were treated without (1) or with $2 \times 10^{-8}$ M Dex (2), 10 µg $ml^{-1}$ of vesnarinone (3), or $2 \times 10^{-8}$ M Dex plus 10 µg $ml^{-1}$ of vesnarinone (4) for 2 days. ppRb; phosphorylated Rb protein, pRb; dephosphorylated Rb protein induce significant changes in the morphology of EBC-1 cells, but Dex-induced morphological changes were slightly enhanced by vesnarinone.

Since alkaline phosphatase activity is a marker for the maturation of type II pneumocytes (Edelson et al, 1988; McCormick et al, 1995), we measured the effect of Dex and vesnarinone on alkaline phosphatase activity in EBC-1, ABC-1 and PC9 cells. Dex significantly induced alkaline phosphatase activity in the cells, as in a previous report that Dex induces alkaline phosphatase activity in A549 cells (McCormick et al, 1995). Vesnarinone alone did not induce enzyme activity in the cells, but did significantly enhance the Dex-induced alkaline phosphatase activity in EBC-1 (Figure 5A), ABC-1 and PC9 cells (Figure 5B).

### Cell cycle analysis of EBC-1 cells treated with vesnarinone and Dex

To understand the nature of the joint action of vesnarinone and Dex on the proliferation of EBC-1 cells, we exposed the cells to 10 µg $ml^{-1}$ of vesnarinone and $2 \times 10^{-8}$ M Dex, and then measured the cell cycle distribution after 4 days (Figure 6). This treatment induced growth arrest of the cells at the $G_0$–$G_1$ phase. There were no appreciable apoptotic cells in a culture incubated with vesnarinone and Dex at 7 days. The $G_1$ arrest in cells treated with vesnarinone plus Dex was more prominent than that in cells cultured in serum-free medium or under confluent conditions (Table 3).

**Table 3** Effects of Dex and vesnarinone on the cell-cycle distribution of EBC-1 cells

| Treatment | Cell cycle distribution (% ± s.d.) | | |
|---|---|---|---|
| | $G_1$ | S | $G_2$+M |
| Rapidly growing | 47.5 ± 4.8 | 38.2 ± 3.5 | 14.3 ± 2.1 |
| Confluent | 62.4 ± 6.5 | 23.5 ± 2.8 | 14.1 ± 1.7 |
| Serum-free | 63.2 ± 5.9 | 21.8 ± 2.3 | 15.0 ± 1.4 |
| Dex | 54.5 ± 5.9 | 26.9 ± 2.4 | 18.6 ± 2.2 |
| Ves | 86.6 ± 8.2 | 2.3 ± 0.4 | 11.1 ± 1.2 |
| Dex+Ves | 97.1 ± 9.4 | 0 | 2.9 ± 0.3 |

EBC-1 cells were incubated with or without 20 µg $ml^{-1}$ of vesnarinone (Ves) in the presence or absence of $2 \times 10^{-8}$ M Dex for 7 days. Cells were cultured at a cell density below $3 \times 10^5$ $ml^{-1}$ in 10% serum-containing medium (rapidly growing cells) or in serum-free medium (serum-free) for 4 days. Means ± s.d. of three determinations.

### Dephosphorylation of Rb protein in EBC-1 cells treated with vesnarinone and Dex

A reasonable hypothesis is that arrest of the cell cycle in $G_1$ was caused by dephosphorylation of Rb protein, whereas the transient $G_2$ block was independent of Rb protein dephosphorylation (Weinberg, 1995). Phosphorylation of Rb protein of EBC-1 cells treated with vesnarinone and Dex was analysed by Western blotting using anti-Rb protein monoclonal antibody (Figure 7). In untreated cells, Rb protein was hyperphosphorylated and migrated slower, while Rb protein was under-phosphorylated and migrated faster in cells treated with vesnarinone and Dex. Phosphorylation began to decrease after 24 h of treatment.

 © Cancer Research Campaign 1999

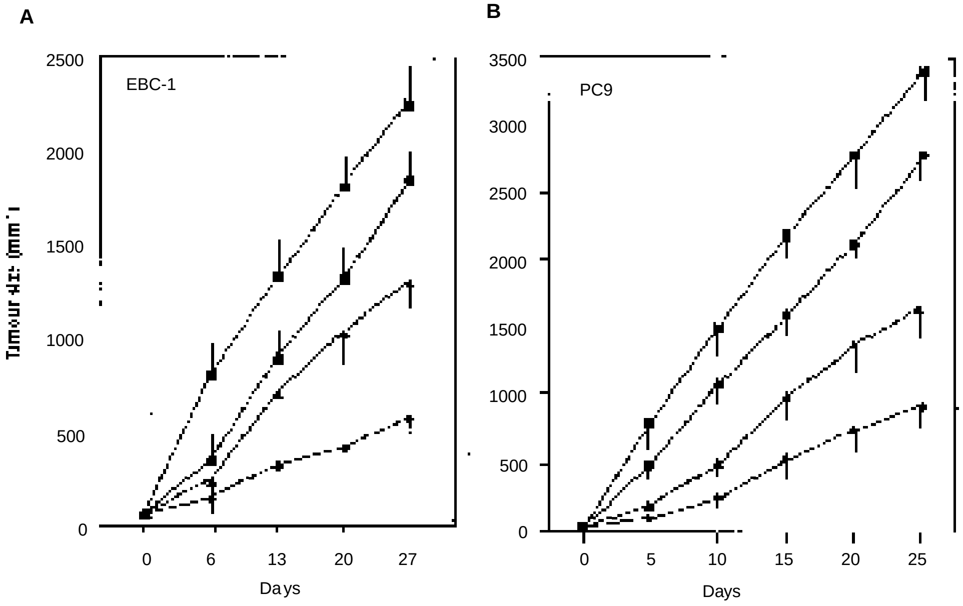

**Figure 8** Effects of prednisolone and vesnarinone on the growth of EBC-1 (**A**) and PC9 (**B**) cells as xenografts. Mice received daily p.o. administration of vesnarinone (▲, ◆) and i.p. injection of prednisolone (●, ◆). ■, untreated mice. Values are means (± s.d.) of 7–10 mice

### Effect of vesnarinone and prednisolone on the in vivo growth of EBC-1 cells as xenografts

The in vitro studies described above suggested that combined treatment with vesnarinone and glucocorticoid should be more effective therapeutically than treatment with vesnarinone or glucocorticoid alone. Although Dex was the most potent steroid tested in the in vitro studies, preliminary results indicated that prednisolone was more effective than Dex in the in vivo experiments. Therefore, we used prednisolone for the further in vivo experiments. The combined treatment significantly inhibited the growth of EBC-1 cells as xenografts (Figure 8). At day 20 after the inoculation of tumour cells, the mean tumour volumes of untreated, prednisolone-, vesnarinone- and prednisolone plus vesnarinone-treated nude mice were 1.82 ± 0.12, 1.32 ± 0.16, 0.98 ± 0.13, and 0.36 ± 0.11 $cm^3$ (± s.d.) respectively. These results indicate that the combination of vesnarinone and prednisolone is more effective therapeutically than vesnarinone or prednisolone alone, and the anti-tumour effect of the combined treatment was statistically significant ($P < 0.01$). The combined treatment was also effective in xenografts of lung carcinoma PC9 cells (Figure 8).

### DISCUSSION

Glucocorticoid receptor is widely distributed in human tissues, but receptor activation and regulation are tissue-specific. Glucocorticoids accelerate the appearance of pulmonary surfactant in developing mammalian fetal lungs (Liggins, 1969; DeLemos et al, 1970; Liggins and Howie, 1972), and hydrocortisone inhibits the rate of mitosis in fetal rabbit lung (Jones et al, 1978). Glucocorticoids have slight growth-inhibitory effects on some NSCLC cells (Kikkawa et al, 1971; Mattern et al, 1985; McLean et al, 1986), suggesting that lung cancer may be influenced by glucocorticoid therapy.

Growth of human lung alveolar carcinoma A549 cells is dose-dependently inhibited by glucocorticoids, and this growth inhibition is associated with an induction of differentiated function, although the differentiation of lung cancer cells has not been well characterized (Kikkawa et al, 1971; Mattern et al, 1985; McCormick et al, 1995). Dex induced morphological changes as well as alkaline phosphatase activity in EBC-1 cells, and the same concentrations were effective in combination with vesnarinone, suggesting that these phenotypic changes were partly associated with the sensitization of EBC-1 cells to vesnarinone. Other glucocorticoids were also effective in inhibiting growth in combination with vesnarinone (Table 1). The growth-inhibitory effect of Dex plus vesnarinone was observed in all of the NSCLC cells tested. A significant incidence of specific, high-affinity receptors for glucocorticoids is present in NSCLC (Chaudhuri et al, 1982; Beattie et al, 1985; Hofmann et al, 1995). The growth of approximately one-third of the NSCLC cell lines was inhibited by Dex, although the growth-inhibitory effect was modest in most of the cell lines (Hofmann et al, 1995). Glucocorticoids can be administered to patients with NSCLC with tolerable side-effects, although the steroids alone have no anticancer effect in lung cancer patients. Glucocorticoids more than additively enhanced vesnarinone-induced growth inhibition. Therefore, treatment with glucocorticoids in combination with vesnarinone may be a strategy for treating lung cancer with low sensitivity to conventional chemotherapy.

Cyclin-dependent kinase-mediated phosphorylation of Rb protein is prevented by cyclin-dependent kinase inhibitors such as p21 (WAF1/CIP1/SDI1) (Weinberg, 1995). Therefore, we examined the expression of p21 mRNA in EBC-1 cells that had been treated with vesnarinone and Dex. Untreated EBC-1 cells expressed a detectable level of p21 mRNA, but this expression was not affected by vesnarinone and/or Dex (data not shown). Similar results were obtained when the expression of p21 protein

*© Cancer Research Campaign 1999*

was analysed by immunoblot. In most NSCLC, p21 protein and RNA were expressed at higher levels than in the corresponding normal tissues, and p21 was expressed independent of changes in p53 gene/protein (Marchetti et al, 1996). These results suggest that the dephosphorylation of Rb protein by vesnarinone and Dex is independent of the induction of p21 cyclin-dependent kinase inhibitor.

Induction of protein phosphatase activity rather than p21 is responsible for chemotherapy-induced Rb dephosphorylation and consequent $G_1$ arrest in human leukaemia cells (Christoffersen et al, 1994; Dou et al, 1995). Rb protein phosphatase may be predominantly responsible for p53-independent Rb dephosphorylation, $G_1$ arrest and apoptosis (Alberts et al, 1993; Ludlow et al, 1993; Christoffersen et al, 1994; Dou et al, 1995). Preliminary experiments indicate that combined treatment with vesnarinone and Dex induces Rb protein phosphatase activity in EBC-1 cells (data not shown), although we cannot rule out the participation of Rb protein kinase inhibitors other than p21.

Solid tumour cells are generally more resistant to anticancer drugs and apoptosis-inducing agents than leukaemia cells. However, the growth-inhibitory effects of vesnarinone were more prominent in solid tumour cells than in leukaemia cells (Sato et al, 1995, 1996). The actions of many anticancer drugs are mediated by the induction of p53 and/or p21, but the growth-inhibitory effects of vesnarinone on EBC-1 cells were independent of p21. Since many lung cancer cells have an impaired p53/p21-signalling system, a p53/p21-independent mechanism would be important for regulating the abnormal growth of lung cancer cells.

Transforming growth factor (TGF)-β inhibits the growth and induces differentiation of several lung and other carcinoma cells (Twardzik et al, 1989; Chakrabarty et al, 1990; Schroy et al, 1990). Glucocorticoids synergistically inhibit the growth of monocytoid leukaemia cells in combination with a low concentration of TGF-β (Kanatani et al, 1996). Vesnarinone, like TGF-β, induces the differentiation of human erythroleukaemia HEL cells (Sato et al, 1996). However, TGF-β production was not induced by vesnarinone and/or Dex in EBC-1 cells, and TGF-β did not have a synergistic effect with Dex in the growth inhibition of EBC-1 cells (data not shown). These results suggest that TGF-β is not involved in the growth inhibition of EBC-1 cells by Dex and vesnarinone.

Vesnarinone and glucocorticoids are non-genotoxic, and clinically available. Vesnarinone has been approved for use as a cardiotonic drug. The serum level of vesnarinone can be obtained up to 30 $\mu g\ ml^{-1}$ (Miyamoto and Sasabe, 1984; Taira et al, 1984). There is no appreciable toxicity of vesnarinone on oral administration, since absorption of the drug is saturated due to the low solubility. On rare occasion granulocytopenia (less than 2%) was observed by vesnarinone in humans, but it is reversible when the administration is stopped. Therapy with glucocorticoids or vesnarinone alone may be effective in only a very limited number of cases of NSCLC, since glucocorticoids only have a marked anti-proliferative effect on A549 cells (Jones et al, 1978). Vesnarinone alone has significant anti-tumour activity against human salivary cancer cells in athymic nude mice (Sato et al, 1995). In human NSCLC cell lines, vesnarinone only had such a significant effect on LK2 cells. However, the combination of vesnarinone and Dex had a significant anti-proliferative effect on all of the lung cancer cell lines tested, and the anti-proliferative effect in vivo was observed without any significant adverse effects. This may be a promising approach to lung cancer therapy which avoids many of the disadvantages of cytotoxic agents.

### ACKNOWLEDGEMENTS

This work was supported in part by Grants for Cancer Research from the Ministry of Education, Science, Sports and Culture of Japan and the Ministry of Health and Welfare of Japan.

### REFERENCES

Alberts AS, Thorburn AM, Shenolikar S, Mumby MC and Feramisco JR (1993) Regulation of cell cycle progression and nuclear affinity of the retinoblastoma protein by protein phosphatases. *Proc Natl Acad Sci USA* **90**: 388–392

Beattie CW, Hansen NW and Thomas PA (1985) Steroid receptors in human lung cancer. *Cancer Res* **45**: 4206–4214.

Chakrabarty S, Fan D and Varani J (1990) Modulation of differentiation and proliferation in human colon carcinoma cells by transforming growth factor β1 and β2. *Int J Cancer* **46**: 493–499.

Chaudhuri PK, Thomas PA, Walker MJ, Briele HA, Gupta TKD and Beattie CW (1982) Steroid receptors in human lung cancer cytosols. *Cancer Lett* **16**: 327–332

Christoffersen J, Smeland EB, Stokke T, Tasken K, Anderson KB and Blomhoff HK (1994) Retinoblastoma protein is rapidly dephosphorylated by elevated cyclic adenosine monophosphate levels in human B-lymphoid cells. *Cancer Res* **54**: 2245–2250

DeLemos RA, Shermeta DW, Knelson JH, Kotas R and Avery ME (1970) Acceleration of appearance of pulmonary surfactant in the fetal lamb by administration of corticosteroids. *Am Rev Respir Dis* **102**: 459–461

Donnadieu N, Paesmans M and Sculier JP (1991) Chemotherapy of non-small lung cancer according to disease extent: a meta-analysis of the literature. *Lung Cancer* **7**: 243–252

Dou QP, An B and Will PL (1995) Induction of a retinoblastoma phosphatase activity by anticancer drugs accompanies p53-independent $G_1$ arrest and apoptosis. *Proc Natl Acad Sci USA* **92**: 9019–9023

Edelson JD, Shannon JM and Mason RJ (1988) Alkaline phosphatase: a marker of alveolar type II cell differentiation. *Am Rev Respir Dis* **138**: 1268–1275

Goto I, Hozumi M and Honma Y (1994) Selective effect of *O*-alkyl lysophospholipids on the growth of a human lung giant cell carcinoma cell line. *Anticancer Res* **14**: 357–362

Goto I, Yamamoto-Yamaguchi Y and Honma Y (1996) Enhancement of sensitivity of human lung adenocarcinoma cells to growth-inhibitory activity of interferon-α by differentiation-inducing agents. *Br J Cancer* **74**: 546–554

Hartwell LH and Kastan MB (1994) Cell cycle control and cancer. *Science* **266**: 1821–1828

Hofmann J, Kaiser U, Maasberg M and Havemann K (1995) Glucocorticoid receptors and growth inhibitory effects of dexamethasone in human lung cancer cell lines. *Eur J Cancer* **31A**: 2053–2058

Jones KL, Anderson NS and Addison J (1978) Glucocorticoid-induced growth inhibition of cells from a human lung alveolar cell carcinoma. *Cancer Res* **38**: 1688–1693

Kanatani Y, Kasukabe T, Hozumi M, Motoyoshi K, Nagata N and Honma Y (1993) Genistein exhibits preferential cytotoxicity to a leukemogenic variant but induces differentiation of a non-leukemogenic variant of the mouse monocytic leukemia Mm cell line. *Leuk Res* **17**: 847–853

Kanatani Y, Kasukabe T, Okabe-Kado J, Hayashi S, Yamamoto-Yamaguchi Y, Motoyoshi K, Nagata N and Honma Y (1996) Transforming growth factor β and dexamethasone cooperatively enhance c-jun gene expression and inhibit the growth of human monocytoid leukemia cells. *Cell Growth Diff* **7**: 187–196

Karp JE and Broder S (1995) Molecular foundations of cancer: new targets for intervention. *Nature Med* **1**: 309–320

Kikkawa Y, Kaibara M, Motoyama EK and Cook CD (1971) Morphologic development of fetal rabbit lung and its acceleration with cortisol. *Am J Pathol* **64**: 423–442

Laemmli UK (1970) Cleavage of structure proteins during the assembly of the head of bacteriophage T4. *Nature* **227**: 680–685

Liggins GC (1969) Premature delivery of fetal lambs infused with glucocorticoids. *Endocrinology* **45**: 515–523

Liggins GC and Howie RNA (1972) Controlled trial of antepartum glucocorticoid treatment for prevention of the respiratory distress syndrome in premature infants. *Pediatrics* **50**: 515–525

Ludlow JW, Glendening CL, Livingston DM and DeCaprio JA (1993) Specific enzymatic dephosphorylation of the retinoblastoma protein. *Mol Cell Biol* **13**: 367–372

 *© Cancer Research Campaign 1999*

Marchetti A, Doglion C, Barbareschi M, Buttitta F, Pellegrini S, Bertacca G, Chella A, Merlo G, Angeletti CA, Palma PD and Bevilacqua G (1996) p21 RNA and protein expression in non-small cell lung carcinomas: evidence of p53-independent expression and association with tumoral differentiation. *Oncogene* **12**: 1319–1324

Mattern J, Klinga K, Rummebaum B and Volm M (1985) Influence of hormone therapy on human lung tumors transplanted into nude mice. *Oncology* **42**: 388–390

McCormick C, Freshney RI and Speirs V (1995) Activity of interferonα, interleukin 6 and insulin in the regulation of differentiation in A549 alveolar carcinoma cells. *Br J Cancer* **71**: 232–239

McLean JS, Frame MC, Freshney RI, Vaughan PFT, Mackie AE and Singer I (1986) Phenotypic modification of human glioma and non-small cell lung carcinoma by glucocorticoids and other agents. *Anticancer Res* **6**: 1101–1106

Miyamoto G and Sasabe H (1984) Pharmacokinetics of a new positive inotropic agent, 3,4-dihydro-6-[4-(3,4-dimethoxybenzoyl)-1-piperazinyl]-2(1H)-quinolinone (OPC-8212), in the rat, rabbit, beagle dog and rhesus monkey. *Arzneimittel-Forschung Drug Res* **34**: 394–402

OPC-8212 Multicenter Research Group (1990) A placebo-controlled, randomized, double-blind study of OPC-8212 in patients with mild chronic heart failure. *Cardiovasc Drug Ther* **4**: 419–426

Sato M, Harada K, Bando T, Shirakami T, Nakashiro K, Yoshida H, Nakai S, Kawai K and Adachi M (1995) Characteristics of antitumor activity of 3,4-dihydro-6-[4-(3,4-dimethoxybenzoyl)-1-piperazinyl]-2 (1H)-quinolinone (Vesnarinone) against a human adenoid squamous carcinoma-forming cell line grown in athymic nude mice. *Cancer Lett* **91**: 1–9

Sato S, Tomoyasu S, Okabe-Kado J, Hozumi M, Tsuruoka N, Nakai S, Adachi M and Honma Y (1996) Induction of differentiation and enhancement of vincristine sensitivity of human erythroleukemia HEL cells by vesnarinone, a positive inotropic agent. *Exp Hematol* **24**: 37–42

Schroy P, Rifkin J, Coffey RJ, Winawer S and Friedman E (1990) Role of transforming growth factor β in induction of colon carcinoma differentiation by hexamethylene bisacetamide. *Cancer Res* **50**: 261–265

Taira N, Endoh M, Iijima T, Satoh K, Yanagisawa T, Yamashita S, Maruyama M, Kawada M, Morita T and Wada Y (1984) Mode and mechanism of action of 3,4-dihydro-6-[4-(3,4-dimethoxybenzoyl)-1-piperazinyl]-2-(1H)-quinolinone (OPC-8212), a novel positive inotropic drug, on the dog heart. *Arzneimittel-Forschung Drug Res* **34**: 35–43

Twardzik DR, Ranchalis JE, McPherson JM, Ogawa Y, Gentry L, Purchio A, Plata E and Todaro GJ (1989) Inhibition and promotion of differentiated-like phenotype of a human lung carcinoma in athymic mice by natural and recombinant forms of transforming growth factor-β. *J Natl Cancer Inst* **81**: 1182–1185

Weinberg RA (1995) The retinoblastoma protein and cell cycle control. *Cell* **81**: 323–330

© Cancer Research Campaign 1999
